# Isolation and Characterization of Novel Reassortant Influenza A(H10N7) Virus in a Harbor Seal, British Columbia, Canada

**DOI:** 10.3201/eid2807.212302

**Published:** 2022-07

**Authors:** Yohannes Berhane, Tomy Joseph, Oliver Lung, Carissa Embury-Hyatt, Wanhong Xu, Paul Cottrell, Stephen Raverty

**Affiliations:** University of Manitoba Department of Animal Science, Winnipeg, Manitoba, Canada (Y. Berhane);; Canadian Food Inspection Agency, Winnipeg (Y. Berhane, O. Lung, C. Embury-Hyatt, W. Xu);; Western College of Veterinary Medicine, Saskatoon, Saskatchewan, Canada (Y. Berhane, S. Raverty);; Ministry of Agriculture, Food and Fisheries, Abbotsford, British Columbia, Canada (T. Joseph, S. Raverty);; University of Manitoba Department of Biological Sciences, Winnipeg (O. Lung);; Fisheries and Aquaculture Management, Vancouver, British Columbia, Canada (P. Cottrell);; University of British Columbia Institute for Oceans and Fisheries, Vancouver (S. Raverty)

**Keywords:** influenza, influenza A virus, avian influenza, H10N7, viruses, whole-genome sequencing, reassortment, harbor seals, British Columbia, Canada

## Abstract

We isolated a novel reassortant influenza A(H10N7) virus from a harbor seal in British Columbia, Canada, that died from bronchointerstitial pneumonia. The virus had unique genome constellations involving lineages from North America and Eurasia and polymerase basic 2 segment D701N mutation, associated with adaptation to mammals.

Influenza A viruses (IAVs) are one of the most consequential pathogens of birds and terrestrial mammals. IAVs (family *Orthomyxoviridae*) are enveloped, segmented, negative-sense, single-stranded RNA viruses, subtyped based on the surface glycoproteins hemagglutinin (H) and neuraminidase (N) embedded in the virus envelope. With the exceptions of H17N10 and H18N11 IAVs in bats, all other existing combinations of H (n = 16) and N (n = 9) subtypes are found in wild aquatic and shore birds, their natural reservoir hosts (*1*).

Phylogenetically, IAVs can be separated into 2 genetically distinct lineages, Eurasia (EA) and North America (NA), because of the fidelity of migratory waterfowl to distinct flyways (*2*). However, in areas where migratory flyways overlap, there are, although rare, IAVs with gene segments from both NA and EA lineages. Surveillance studies in Alaska (United States) and Newfoundland (Canada) documented a preponderance of IAVs with whole-genome segments from EA lineage viruses and only rarely, reassortant IAVs with EA and NA gene segment constellations (*3*,*4*). In 2014, along the Pacific flyway corridor in Canada and the United States, goose/Guangdong/1/96 (Gs/GD) lineage H5N8 virus was detected in wild waterfowl (*5*). The Gs/GD virus reassorted with NA lineage IAVs, subsequently resulting in devastating losses among commercial poultry flocks in Canada and the United States.

Wild bird–origin IAVs can breach the host species barrier and cause outbreaks involving several terrestrial mammals. After crossing the species barrier, some IAVs can become established in new hosts and circulate independently of their reservoir hosts. Different species of marine mammals are susceptible to IAV infection through exposure at haul-out sites, where they might comingle with wild birds, infected sympatric marine mammal species, terrestrial wildlife, including mink and river otters, and through direct exposure to infected humans or waterfowl in rehabilitation facilities (*6*). Previous outbreaks and individual case reports have shown that seals are susceptible to and have died as a result of infection with H10N7, H3N8, H7N7, H4N6, and other IAVs (*6*).

## The Study

**Figure 1:**
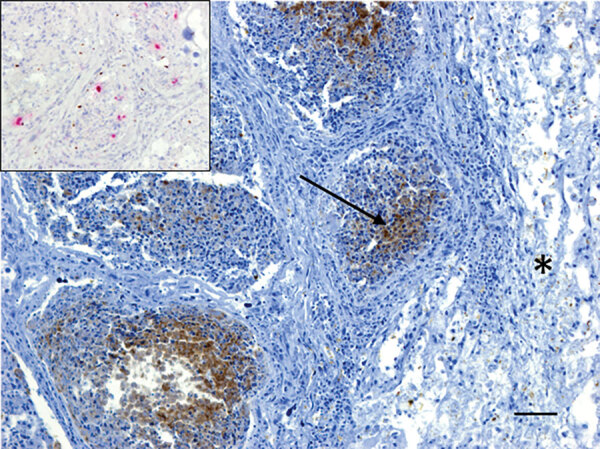
Immunohistochemistry testing for influenza A virus antigen in lung tissue of an adult male harbor seal, British Columbia, Canada. Viral antigen (arrow) was detected by immunohistochemistry within the bronchiolar-associated lymphoid tissue but not in adjacent lung parenchyma (*). Viral RNA could also be detected in bronchiolar-associated lymphoid tissue by in situ hybridization (inset, pink areas). Scale bar = 50 μm.

A live adult male harbor seal weighing 49 kg washed ashore at Combers Beach near Tofino, British Columbia (BC), Canada, but died before he could be captured alive. On June 1, 2021, the seal was brought to the Animal Health Center Laboratory (Abbotsford, BC, Canada) for necropsy. The seal was in moderate body and fair postmortem condition. The most notable findings from gross examination were serosanguinous fluid within the thoracic cavity and focally extensive hemorrhage and edema from the skin to the visceral pleura along the right midlateral aspect of the thorax, with focal visceral to parietal pleural adhesion. Histopathology revealed necrotizing bronchitis and bronchiolitis, peribronchiolar lymphoid hyperplasia, alveolar histiocytosis, and perivascular lymphoplasmacytic cuffing. Immunohistochemistry testing identified IAV viral antigen within the bronchiolar-associated lymphoid tissue of 2 bronchioles in more severely affected areas of the lungs and in situ hybridization confirmed IAV RNA (Figure 1). Aerobic cultures of the lungs, hilar lymph node, brain, intercostal skeletal muscle, and small intestine yielded variable mixed growth of *Streptococcus phocae* and *Serratia liquefaciens* with no fungal growth from the lung. PCR testing of pooled tissues proved positive for consensus influenza virus and mollicutes (we confirmed *Mycoplasma* spp., but were unable to speciate) and negative for canine distemper virus.

We extracted RNA samples from the hilar lymph node, thymus, spleen, and lungs, and thoracic fluid tested positive on a real-time reverse transcription PCR assay based on IAV matrix (M) genes, as described elsewhere (*7*). We isolated all IAV PCR-positive samples in embryonated specific pathogen–free chicken eggs from lung tissue samples only. We amplified all 8 viral gene segments from original lung specimens and isolates as described elsewhere (*8*), then purified amplified reverse transcription PCR products using a QIAGEN QIAquick PCR purification kit (https://www.qiagen.com) and determined the concentration of the amplicons used for sequencing using an Invitrogen Qubit dsDNA BR assay kit (https://www.thermofisher.com) on the Qubit Fluorometer. We performed library prep using Illumina Nextera XT Library preparation kit (https://www.illumina.com) and sequencing using Illumina MiSeq, as described elsewhere (*9*). We assembled IAV full genome segments using DNASTAR SeqMan NGen software version 15.3.0 (https://www.dnastar.com).

**Figure 2:**
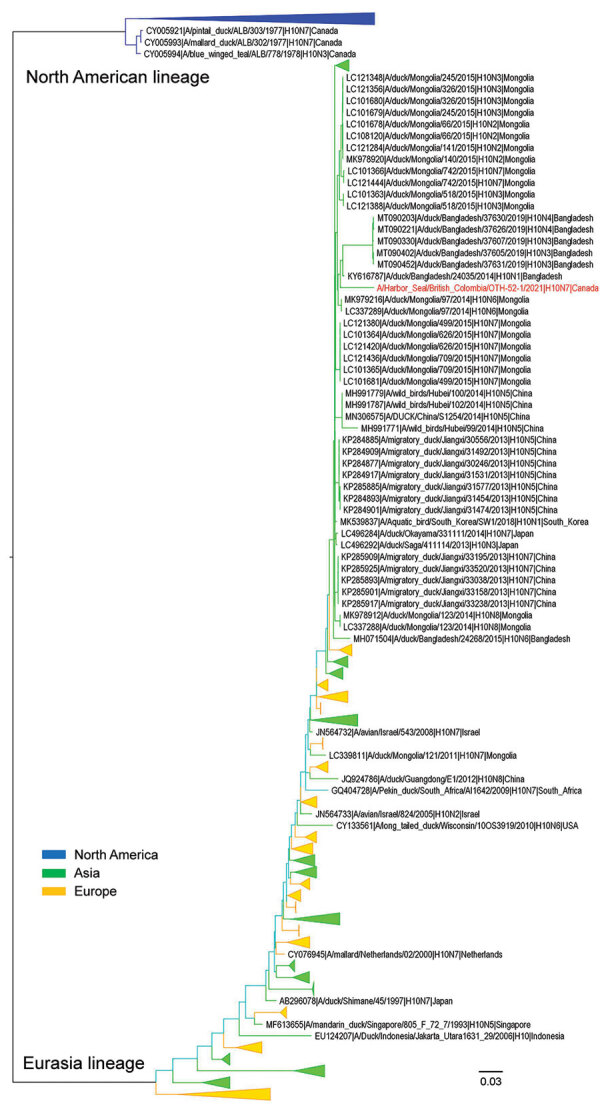
Maximum-likelihood phylogenetic tree of influenza A virus subtype H10 hemagglutinin gene from an adult male harbor seal, British Columbia, Canada (red text), and reference sequences. Phylogenetic analyses were based on the full-length nucleotide sequence of the hemagglutinin gene of strains representing the H10 subtype (n = 1,512). The evolutionary relationship was inferred using RAxML (https://github.com/stamatak/standard-RAxML) based on the general time-reversible model with 1,000 bootstrap replicates. For purposes of clarity, some clades are collapsed, and colors are assigned to indicate the origin of the gene: blue for North America, green for Asia, and yellow for Europe. The tree was drawn to scale; branch lengths are measured in number of substitutions per site.

Full genome sequences of the virus obtained from the original lung tissue samples and isolates from lungs were 100% identical. Further analysis demonstrated that 4 gene segments, polymerase basic (PB) 1 and 2, polymerase acidic (PA), and M segments, originated from unknown NA lineage IAVs of wild bird origin. The remaining 4 gene segments, H, N, nucleoprotein (NP), and nonstructural (NS) were derived from EA lineage IAVs. We designated the virus A/harbor seal/British Colombia/OTH-52-1/2021(H10N7) and deposited sequences from the original sample and isolates into the National Center for Biotechnology Information genome database (https://www.ncbi.nlm.nih.gov/genome) under accession nos. OL336415 (PB2), OL336416 (PB1), OL336417 (PA), OL336418 (H), OL336419 (NP), OL336420 (N), OL336421 (M), and OL336422 (NS). We compared percentage similarity between all 8 gene segments and their associated proteins and closest matches in GenBank (Table). Phylogenetic analysis demonstrated that the H gene of the virus clades with EA lineage IAVs was circulating in wild birds (Figure 2).

**Table:** Similarity of all 8 gene segments and their associated proteins for influenza A(H10N7) virus from a harbor seal in British Columbia, Canada, to closest matches in GenBank*

Segment	Similarity† (%)	Closest match	GenBank accession no.	Lineage
PB2	2,225/2,280 (97.59)	A/ruddy turnstone/New Jersey/AI13-2822/2013	MH500897	North America
PB1	2,240/2,274 (98.50)	A/Mallard/WA/AH0042257S.2.A/2015(H6N8)	MN254524	North America
PA	2,117/2,151 (98.42)	A/blue-winged teal/Texas/AI11-3220/2011(H3N8)	CY205900	North America
H	1,613/1,686 (95.67)	A/duck/Bangladesh/24035/2014 (H10N1)	KY616787	Eurasia
NP	1,467/1,497 (98.00)	A/duck/Hokkaido/W90/2007(H10N7)	LC121485	Eurasia
N	1,372/1,416 (96.89)	A/duck/Mongolia/742/2015(H10N7)	LC121446	Eurasia
M	973/982 (99.08)	A/Mallard duck/Alberta/486/2019(H1N1)	MT624434	North America
NS1/NEP	821/844 (97.27)	A/duck/Hokkaido/56/2017(H12N2)	MK592497	Eurasia
*H, hemagglutinin; M, matrix; n, neuraminidase; NP, nucleoprotein; NS1/NEP, nonstructural protein 1/nuclear export protein; PA, acidic polymerase; PB, basic polymerase. †Nucleotide matches at identical sites.				

Further analysis of the virus genome revealed that the H10N7 virus carries a glutamic acid (E) residue at the aa 627 site of PB2 determined to be associated with decreased replication in mammal cells using growth kinetics and with decreased virulence in mice using lethal dose challenge (*10*,*11*). We also observed an aspartic acid substitution at site aa 701, which has been associated with systemic replication and mortality in mice (*11*,*12*). In addition, this residue substitution in guinea pigs conferred efficient replication of the virus in the nose, trachea, and lungs (*11*). Key amino acid changes were also observed in NP and M segments. The presence of lysine at the aa 319 site in the NP gene segment has been implicated in species adaptation (*12*) and substitution in the A/seal/Mass/1/1980(H7N7) backbone conferred increased binding to importin alpha1. In the M protein, aspartate at aa 30 and alanine at aa 215 sites also increased virulence as indicated by the decreased postexposure survival rate in mice (*13*).

## Conclusions

In our study of a harbor seal infected with a novel reassortment H10N7 IAV containing a unique constellation of NA and EA lineage gene segments, we found no conclusive evidence of where and when the reassortment occurred. However, the seal was recovered at a location within the Pacific avian flyway, where Gs/GD lineage H5N8 virus was detected in 2014 and later reassorted with NA lineage viruses to create novel reassortant H5N2 and H5N1 IAVs that were responsible for large outbreaks in domestic poultry in Canada and the United States (*5*). Harbor seals share habitat with seabirds and shorebirds, and the seal in our study may have been infected through spillover directly from infected birds as happened in cases reported elsewhere (*6*,*8*). In previous outbreaks in seals, the proximate cause of death was attributed to secondary or opportunistic *Mycoplasma* spp. (*14*), which were also detected in this case, or bacterial infection. Finally, because novel IAVs in marine mammals have been shown to be potentially zoonotic (*15*), IAV circulation among seals could have important public health implications for humans and other mammals.
